# Severe Post-spinal Bezold-Jarisch Reflex During Emergency Caesarean Delivery Unmasking Wolff-Parkinson-White Pattern

**DOI:** 10.7759/cureus.101966

**Published:** 2026-01-21

**Authors:** Ryan Glaser, Kemishka Pillay, Jeffrey R Bolon, Yuvishkah Harryprasadh

**Affiliations:** 1 Anaesthesiology, University of Witwatersrand, Johannesburg, ZAF; 2 Anaesthesia, Charlotte Maxeke Johannesburg Academic Hospital (CMJAH), Johannesburg, ZAF; 3 Emergency Medicine, Port Macquarie Base Hospital, Port Macquarie, AUS; 4 Medicine, Chris Hani Baragwanath Academic Hospital, Johannesburg, ZAF

**Keywords:** arrhythmias, bezold-jarisch reflex, bradycardia, caesarean section, ephedrine, hypotension, phenylephrine, spinal anaesthesia, vasopressors, wolff-parkinson-white syndrome

## Abstract

Spinal anaesthesia for caesarean delivery commonly causes maternal hypotension due to sympathetic blockade and may occasionally progress to profound bradycardia with haemodynamic collapse when venous return is markedly reduced. We report the case of a 34-year-old gravida 3 para 2 woman at 36 weeks of gestation who underwent emergency caesarean delivery under single-shot spinal anaesthesia. Shortly after the onset of the block, she developed significant hypotension, which rapidly progressed to profound hypotension with marked bradycardia, producing a clinical picture consistent with the Bezold-Jarisch reflex (BJR). After stabilisation, transient rhythm irregularity with intermittent ectopy was observed, without sustained tachyarrhythmia or supraventricular tachyarrhythmia. The rhythm disturbance was benign and self-limited. Postoperatively, a 12-lead electrocardiogram demonstrated a previously undiagnosed Wolff-Parkinson-White (WPW) pattern, and cardiology follow-up was arranged.

This case highlights (i) the frequency and clinical significance of spinal anaesthesia-induced hypotension during caesarean delivery, (ii) the potential for bradycardic-hypotensive episodes consistent with BJR or vasovagal physiology when venous return is compromised, and (iii) how adrenergic and anticholinergic rescue therapy (ephedrine and atropine), together with physiological stress, may unmask underlying cardiac conduction abnormalities such as WPW.

Current obstetric vasopressor guidelines emphasise proactive blood pressure maintenance, typically using a prophylactic phenylephrine infusion initiated immediately after intrathecal injection in combination with crystalloid co-loading, with rescue vasopressors titrated according to maternal haemodynamics. For newly identified WPW, contemporary recommendations support risk stratification, including assessment for intermittent pre-excitation, and consideration of electrophysiological evaluation and catheter ablation when clinically indicated.

## Introduction

Spinal anaesthesia for caesarean delivery is commonly performed but is frequently complicated by maternal hypotension due to sympathetic blockade [1-4]. In contemporary practice, vasopressors are required in a large proportion of spinal anaesthetics for caesarean delivery, reflecting both the high incidence of hypotension and the emphasis on active prevention and early treatment [3]. Maternal hypotension is clinically important because uteroplacental blood flow lacks autoregulation and is therefore dependent on perfusion pressure, making sustained hypotension undesirable for both maternal well-being and foetal oxygen delivery [4]. Even brief maternal hypotension was associated with significantly higher umbilical arterial (H⁺) and base deficit (i.e., greater neonatal acidaemia) compared with normotensive controls, albeit still within normal limits [5]. Expert guidance recommends strategies that combine positioning to reduce aortocaval compression, fluid co-loading, and prompt vasopressor therapy to maintain systolic arterial pressure near baseline values during spinal anaesthesia for caesarean delivery [1,2,4].

While most episodes of spinal hypotension are readily treated, severe events may be accompanied by marked bradycardia and vasodilation consistent with reflex physiology, such as the Bezold-Jarisch reflex (BJR) and vasovagal mechanisms, particularly when venous return is reduced [3,6]. These episodes may be under-recognized, yet are clinically significant because delayed recognition can allow a seemingly stable period to deteriorate to the first overt signs of collapse within minutes(on average, <2 min after the last apparently adequate assessment during spinal anaesthesia), so intervention to restore preload and heart rate must be immediate to prevent maternal collapse [3,6]. 

Wolff-Parkinson-White syndrome (WPW) is characterized by ventricular pre-excitation due to an accessory atrioventricular pathway, producing classic electrocardiographic findings of a short PR interval, delta wave, and widened QRS complex [7-10]. Many individuals remain asymptomatic, and WPW may only be detected incidentally or after perioperative physiological stress, adrenergic stimulation, or pharmacologic triggers [7-10]. We report a case of severe spinal anaesthesia-associated hypotension followed by profound bradycardia during emergency caesarean delivery, with subsequent identification of previously undiagnosed WPW pattern, and discuss the management in light of obstetric vasopressor consensus statements as well as WPW risk stratification guidance.

This case uniquely links a rapid post-spinal progression from hypotension to profound bradycardic-hypotensive collapse consistent with Bezold-Jarisch/vasovagal physiology, with subsequent identification of previously undiagnosed WPW pattern on postoperative ECG. Key learning points are to manage caesarean spinal haemodynamics proactively (optimise venous return and initiate early vasopressor therapy), escalate promptly when bradycardia accompanies hypotension, and obtain a 12-lead ECG after perioperative rhythm disturbance to detect occult pre-excitation and guide follow-up.

## Case presentation

A 34-year-old gravida 3 para 2 woman at 36 weeks of gestation presented in labour for emergency caesarean delivery after two previous caesarean births. She had no known comorbidities, no drug allergies, and no prior anaesthetic complications. She reported no palpitations, syncope, chest pain, or exercise intolerance, and there was no known family history of cardiac disease. Antenatal care was uncomplicated, and no preoperative ECG had been performed.

In theatre, standard monitoring was applied (including non-invasive blood pressure measurement, continuous electrocardiography, and pulse oximetry), consistent with basic monitoring standards for anaesthesia [10]. Two large-bore intravenous cannulas were inserted.

Single-shot spinal anaesthesia was performed at L3-L4, with hyperbaric bupivacaine 11 mg (0.5%) and fentanyl 10 µg. The patient was positioned supine with left uterine displacement to reduce aortocaval compression.

The heart rate initially remained between 60 and 90 bpm; however, shortly after establishment of the spinal block, it abruptly fell to marked bradycardia (heart rate (HR) 44 bpm) with concurrent severe hypotension (BP 57/34; MAP 41), consistent with a time-sensitive deterioration pattern. Rapid crystalloid resuscitation with Ringer’s lactate was commenced via both intravenous lines as part of supportive management. Ephedrine (10 mg) was administered in bolus doses to restore arterial pressure; phenylephrine was available but was not used because the HR was trending down toward the <60 bpm range, and a vasopressor with β-adrenergic activity was preferred to support both blood pressure and chronotropy. Despite these measures, the patient subsequently developed profound hypotension with marked bradycardia, associated with nausea and lightheadedness. Atropine (200 mcg) was administered, with prompt improvement in both HR and blood pressure, supporting a predominantly vagal mechanism at that time.

**Figure 1:**
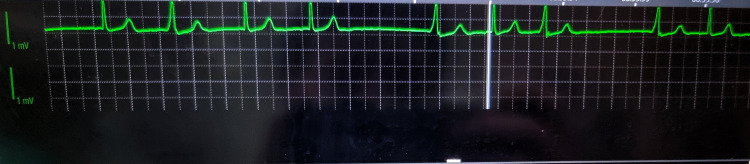
Intraoperative ECG rhythm strip showing transient ectopy after post-spinal haemodynamic collapse Continuous intraoperative ECG monitor tracing (single lead) recorded during the recovery phase after severe post-spinal hypotension with bradycardia. The rhythm is predominantly sinus with regular P-waves preceding most QRS complexes and narrow QRS morphology, consistent with supraventricular activation. Premature ectopic beats present (early QRS with shortened coupling interval), producing brief cycle-length irregularity without progression to sustained tachyarrhythmia. Vertical monitor markers/artefacts are visible and are not part of the cardiac rhythm. The 1 mV calibration marker is shown on the left; paper/grid background reflects monitor display scaling. The strip is de-identified and included to document the transient rhythm disturbance observed perioperatively.

**Figure 2:**
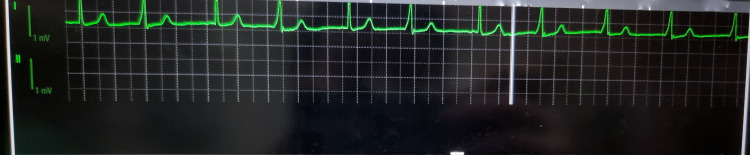
Intraoperative ECG rhythm strip demonstrating sinus rhythm during recovery phase Continuous intraoperative ECG monitor tracing (one displayed channels) recorded after haemodynamic stabilisation following severe post-spinal hypotension with bradycardia, followed by intermittent ectopic beats and brief irregularity (Figure 1). The rhythm is predominantly sinus with respiratory variation, without sustained supraventricular or ventricular tachyarrhythmia. Calibration markers (1 mV) are shown on the left. The trace is included to document the transient rhythm disturbance observed perioperatively.

Following haemodynamic recovery, a brief period of rhythm irregularity with intermittent ectopic beats was observed on continuous ECG monitoring without sustained tachyarrhythmia (Figure 1). The rhythm returned to baseline once intravascular volume was restored and haemodynamic stability achieved. Caesarean delivery proceeded without further complication, and a healthy neonate was delivered (Figure 2).

**Figure 3:**
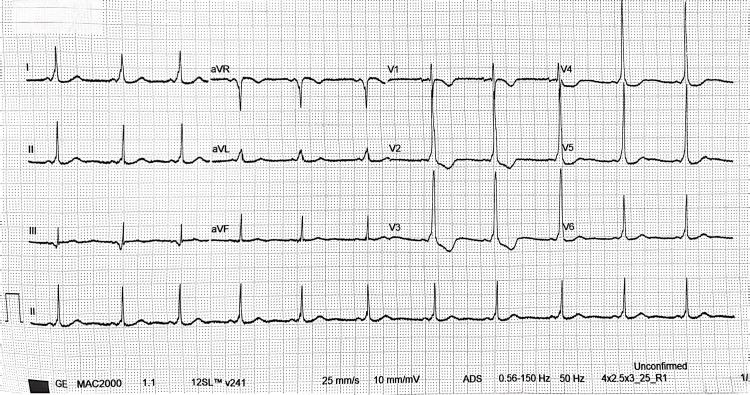
Postoperative 12-lead electrocardiogram demonstrating ventricular pre-excitation consistent with Wolff-Parkinson-White pattern Standard 12-lead ECG obtained after the intraoperative episode of severe post-spinal hypotension with bradycardia. The tracing demonstrates sinus rhythm, with features of ventricular pre-excitation, including a short PR interval, slurred initial upstroke of the QRS complex (delta wave), and widened QRS complexes, consistent with a Wolff-Parkinson-White pattern. The ECG is presented to support the post-event diagnosis and subsequent cardiology referral.

Postoperatively, the patient was admitted to obstetric high care for monitoring. Given the severity of the bradycardic episode and the transient rhythm disturbance, a 12-lead ECG was obtained, which demonstrated a short PR interval with delta waves and a widened QRS complex, consistent with WPW syndrome (Figure 3). Cardiology consultation confirmed the diagnosis, and the patient was scheduled for outpatient follow-up for further assessment and counselling. Single-lead intraoperative ECG limits definitive arrhythmia classification, reinforcing why postoperative 12-lead ECG was appropriate.

## Discussion

Spinal hypotension in caesarean delivery and why it matters

Maternal hypotension during spinal anaesthesia for caesarean delivery is common and clinically relevant, with significant maternal symptoms (including nausea) and potential foetal implications when uteroplacental perfusion pressure falls [1-4]. In South African obstetric practice, the high frequency of hypotension has been emphasized, with vasopressors reportedly required in a substantial proportion of cases, illustrating that “routine” spinal anaesthesia still demands active haemodynamic management [3]. Consensus guidance and enhanced recovery recommendations both support routine prophylaxis and early treatment rather than reactive rescue alone [1,4].

From a physiological standpoint, spinal anaesthesia reduces systemic vascular resistance and venous return through sympathetic blockade, and these effects can be amplified by aortocaval compression in the supine pregnant patient [1,2,4]. Because uteroplacental blood flow lacks autoregulation, it depends directly on uterine perfusion pressure and inversely on uterine vascular resistance, making maternal blood pressure a key determinant of placental perfusion during neuraxial anaesthesia [4]. Therefore, maintaining systolic pressure close to baseline is a central management target in obstetric spinal anaesthesia [1,2].

Profound bradycardia with hypotension: reflex physiology and the Bezold-Jarisch framework

A subset of patients experience marked bradycardia with hypotension after spinal anaesthesia, a pattern often discussed in relation to vasovagal syncope and the BJR [3,6]. In the perioperative context, this pattern has been described as a cardiac depressor reflex involving bradycardia, vasodilation, and hypotension, and it is clinically associated with bradycardia occurring alongside hypotension [3,6]. Reduced venous return is a key precipitating factor for such episodes, and higher neuraxial block levels and healthy physiology have been associated with susceptibility in broader perioperative populations [3,6].

In this case, the sequence of early hypotension followed by nausea and profound bradycardia is consistent with severe reduction in venous return after spinal anaesthesia, potentially exacerbated by aortocaval compression and relative hypovolaemia from labour-associated physiological shifts [1-4,6]. Though the clinical picture is best viewed as a spectrum of reflex-mediated cardiovascular depression, with the BJR and vasovagal responses sharing overlapping vagal predominance and often coexisting rather than representing mutually exclusive mechanisms.

Nausea and lightheadedness are recognized clinical features accompanying spinal hypotension and reflex bradycardia, and their appearance should prompt immediate haemodynamic correction [1,3,4,6]. The prompt response to atropine is consistent with a major vagal component to the bradycardia [6,11].

Preventing and treating spinal hypotension: fluid co-loading and vasopressors

Current guidance supports a multimodal approach to spinal hypotension prevention, including left uterine displacement, rapid crystalloid co-loading, and early vasopressor use [1,4]. In enhanced recovery recommendations for caesarean delivery, prophylactic phenylephrine infusion started immediately after intrathecal injection is supported by current evidence, and crystalloid co-loading (approximately 1 L administered rapidly after spinal injection) is favoured over preload [4]. These recommendations align with broader consensus statements emphasizing proactive haemodynamic maintenance and the use of vasopressors with predominantly direct alpha-adrenergic activity (such as phenylephrine) as primary agents, with fluids as complementary therapy rather than sole treatment [1,4].

Phenylephrine has been extensively discussed as a first-line vasopressor in obstetric spinal anaesthesia because it increases systemic vascular resistance and supports arterial pressure [1,2]. Practical reviews emphasize titrated prophylactic infusions and/or bolus regimens with attention to balancing blood pressure control against reflex bradycardia [2]. In comparative obstetric vasopressor research, phenylephrine infusion strategies have been studied against bolus strategies, with outcomes including maternal haemodynamics and neonatal acid-base status [12]. Choudhary and Bajaj compared reactive phenylephrine 50 µg boluses with a prophylactic phenylephrine infusion (50 µg/min) started immediately after spinal anaesthesia [12]. The infusion strategy provided tighter control and reduced nausea/vomiting, while neonatal outcomes (Apgar and umbilical artery pH/acidosis) were similar between groups [12].

Ephedrine remains widely used, particularly when hypotension coexists with bradycardia, because of its mixed alpha- and beta-adrenergic effects and ability to augment HR and cardiac output [1,2]. Ephedrine’s indirect β-agonist activity, particularly via β₁ receptor stimulation, can increase myocardial excitability and may promote ectopy in susceptible patients. However, obstetric vasopressor literature has long compared ephedrine with phenylephrine with respect to maternal haemodynamics and foetal acid-base outcomes [1,2]. Accordingly, many contemporary protocols prioritize alpha-agonist vasopressors (phenylephrine or norepinephrine) for routine prophylaxis and treatment, reserving ephedrine or anticholinergics for specific haemodynamic profiles such as hypotension with bradycardia [1,2,4].

Why this patient may have deteriorated despite early rescue

Although hypotension is expected after spinal anaesthesia, severe progression can occur when compensatory autonomic responses are insufficient or when venous return is critically reduced [3]. The prediction literature highlights the importance of autonomic function and notes that while sympathectomy is a dominant mechanism, the ability to compensate depends on the autonomic nervous system, and some patients may be less able to maintain blood pressure [3]. The same review describes reflex bradycardic-hypotensive phenomena under the BJR umbrella and emphasizes that reliable prediction of catastrophic events in obstetric spinal anaesthesia remains limited [3].

In this case, initial hypotension without bradycardia followed by profound bradycardia suggests an evolving pathophysiology, where early sympathectomy-related vasodilation may have progressed to a state of markedly reduced preload and reflex vagal activation [3,4,6]. Ephedrine boluses may transiently support pressure but can be insufficient if venous return remains compromised, underscoring the importance of simultaneous measures to restore preload (uterine displacement, rapid co-load, and prompt vasopressor titration) [1-4,6]. Because reflex-mediated cardiovascular depression is triggered by reduced cardiac venous return, vasopressors given without restoring preload may worsen the physiology in susceptible patients, so treatment should prioritise restoration of venous return and correction of absolute blood volume deficits, preventing a paradoxical worsening. The rapid improvement after atropine supports a dominant vagal component at the time of collapse but does not exclude ongoing low preload as the driving trigger [3,4,6].

WPW syndrome: relevance to obstetric anaesthesia and perioperative triggers

WPW syndrome is an atrioventricular pre-excitation condition caused by an accessory pathway that permits early ventricular activation and can support re-entrant tachyarrhythmias [7-10]. Diagnostic ECG features include a short PR interval, delta wave, and widened QRS complex, all of which were present postoperatively in this patient [7-10].

WPW pattern refers to ECG evidence of ventricular pre-excitation (short PR with delta wave/widened QRS) in the absence of arrhythmia-related symptoms, whereas WPW syndrome denotes pre-excitation plus documented or symptomatic tachyarrhythmias attributable to an accessory pathway [10]. Many individuals with pre-excitation are asymptomatic, and risk stratification focuses on identifying pathways capable of rapid anterograde conduction, which can be inferred using non-invasive markers such as intermittent loss of the delta wave on serial ECGs or ambulatory monitoring [10].

Classic electrophysiology teaching emphasises that intermittent pre-excitation (sudden loss of the delta wave with QRS normalization and PR prolongation) is generally associated with a longer accessory pathway refractory period and lower-risk characteristics during atrial fibrillation, making it a useful non-invasive low-risk marker when correctly identified [10]. Contemporary approaches to asymptomatic pre-excitation similarly emphasise clinical assessment, ECG pattern evaluation, and selection of patients for electrophysiology study, along with potential catheter ablation based on risk features and patient context [8,10].

In the perioperative setting, adrenergic surges, haemodynamic instability, and pharmacologic agents can increase ectopy and provoke arrhythmias in susceptible patients, which is relevant when WPW is present but previously unrecognized [7-9]. Although this patient did not develop sustained tachyarrhythmia intraoperatively, the transient ectopy observed after atropine/ephedrine and physiological stress underscores the value of early post-event 12-lead ECG when unexplained rhythm disturbances occur during obstetric neuraxial anaesthesia [7-11].

Immediate arrhythmia considerations when WPW is suspected

Guideline-based management of supraventricular tachycardia (SVT) and pre-excitation emphasizes that the acute approach depends on rhythm type and haemodynamic stability and that certain AV nodal-blocking agents may be hazardous in the setting of pre-excited atrial fibrillation because they can facilitate conduction over the accessory pathway [7,9]. Accordingly, unstable tachyarrhythmias require prompt synchronized cardioversion, while stable presentations require rhythm-specific therapy consistent with SVT guidance [7,9]. In this case, absence of sustained tachyarrhythmia meant that immediate definitive antiarrhythmic therapy was not required, but identification of WPW altered counselling, follow-up planning, and future anaesthetic risk framing [7-10].

Practical lessons for obstetric teams

First, spinal hypotension should be anticipated and prevented with a protocolized bundle (including left uterine displacement, rapid crystalloid co-load, and early vasopressor therapy) because hypotension remains common even in healthy parturients [1,3,4]. Second, profound bradycardia with hypotension after spinal anaesthesia should be treated as an emergency consistent with vasovagal/BJR physiology, prioritizing restoration of venous return and timely anticholinergic/vasopressor treatment [3,6]. Third, when perioperative rhythm irregularity is observed, especially following severe haemodynamic events, obtaining a 12-lead ECG is a low-burden intervention that can reveal pre-excitation and enable risk stratification and follow-up [7-10].

Finally, enhanced recovery guidance reinforces that many intraoperative symptoms such as nausea are commonly driven by spinal hypotension, which can often be mitigated by optimized haemodynamic management and complementing standard antiemetic strategies [4,13,14]. Evidence also highlights 5-HT3 antagonists such as ondansetron as adjuncts to reduce spinal anaesthesia-associated hypotension and related symptoms, although vasopressor-centred prevention remains foundational [13,14].

This case brings to light a potentially under-utilised adjunct for attenuating spinal-anaesthesia-induced hypotension and reflex bradycardia, namely, prophylactic 5-HT3 antagonism with ondansetron [13,14]. Mechanistically, ondansetron may blunt serotonin-mediated activation of the BJR, which is implicated in the vasodilation/bradycardia cascade seen after neuraxial sympathectomy [13,14].

Clinically, a contemporary systematic review and meta-analysis of randomised trials found that ondansetron prophylaxis reduced the incidence of hypotension, reduced bradycardia, and decreased vasopressor requirements after spinal anaesthesia, supporting its role as a haemodynamic-stabilising strategy [13,14].

More recently, a double-blind randomised trial in caesarean delivery demonstrated that prophylactic 4 mg or 8 mg ondansetron improved systolic blood pressure stability and that 8 mg specifically reduced the incidence of post-spinal hypotension compared with placebo [14].

In a parturient subsequently shown to have WPW pattern, any intervention that reduces the depth/duration of hypotension and thereby limits the need for repeated rescue boluses of sympathomimetics may be particularly attractive. Large adrenergic swings can plausibly increase the likelihood of transient rhythm instability in susceptible patients.

Accordingly, while vasopressors and volume optimisation remain central, pre-spinal ondansetron (e.g., 4-8 mg IV) could be considered as an adjunct within a bundled prevention strategy for selected caesarean patients, especially where severe hypotension/bradycardia would be poorly tolerated or where minimising rescue vasopressor exposure is desirable [13,14].

## Conclusions

Spinal anaesthesia-associated hypotension during caesarean delivery is common and requires proactive prevention and rapid treatment to protect maternal comfort and uteroplacental perfusion. When hypotension is accompanied by profound bradycardia and nausea, reflex mechanisms such as vasovagal/BJR physiology should be considered, then managed urgently with restoration of venous return and appropriate anticholinergic/vasopressor therapy. New postoperative identification of WPW following intraoperative rhythm irregularity highlights the value of early ECG assessment after severe haemodynamic events and enables appropriate counselling as well as outpatient risk stratification.
